# Preparation, Characterization, and Drug Delivery of Hexagonal Boron Nitride-Borate Bioactive Glass Biomimetic Scaffolds for Bone Tissue Engineering

**DOI:** 10.3390/biomimetics8010010

**Published:** 2022-12-26

**Authors:** Mertcan Ensoylu, Aylin M. Deliormanlı, Harika Atmaca

**Affiliations:** 1 Department of Metallurgical and Materials Engineering, Manisa Celal Bayar University, 45140 Yunusemre, Manisa, Turkey; 2 Department of Biology, Manisa Celal Bayar University, 45140 Yunusemre, Manisa, Turkey

**Keywords:** bioactive glass, hBN nanoparticles, biomimetic, scaffolds, drug delivery

## Abstract

In this study, biomimetic borate-based bioactive glass scaffolds containing hexagonal boron nitride hBN nanoparticles (0.1, 0.2, 0.5, 1, and 2% by weight) were manufactured with the polymer foam replication technique to be used in hard tissue engineering and drug delivery applications. To create three-dimensional cylindrical-shaped scaffolds, polyurethane foams were used as templates and covered using a suspension of glass and hBN powder mixture. Then, a heat treatment was applied at 570 °C in an air atmosphere to remove the polymer foam from the structure and to sinter the glass structures. The structural, morphological, and mechanical properties of the fabricated composites were examined in detail. The in vitro bioactivity of the prepared composites was tested in simulated body fluid, and the release behavior of gentamicin sulfate and 5-fluorouracil from glass scaffolds were analyzed separately as a function of time. The cytotoxicity was investigated using osteoblastic MC3T3-E1 cells. The findings indicated that the hBN nanoparticles, up to a certain concentration in the glass matrix, improved the mechanical strength of the glass scaffolds, which mimic the cancellous bone. Additionally, the inclusion of hBN nanoparticles enhanced the in vitro hydroxyapatite-forming ability of bioactive glass composites. The presence of hBN nanoparticles accelerated the drug release rates of the system. It was concluded that bioactive glass/hBN composite scaffolds mimicking native bone tissue could be used for bone tissue repair and regeneration applications.

## 1. Introduction

Bone tissue may be deformed and unable to function because of aging, disease, trauma, or injury. Today, new methods are being developed for the treatment and regeneration of damaged bone tissue [1]. Ceramic, polymer, and metal-based biomaterials can be utilized in bone tissue engineering applications. Another type of biomedical material utilized in the same application area is bioactive glass. Bioactive glasses are materials that can react with physiological fluids and bond to the bone surface by forming hydroxyapatite on their surface [2]. They exhibit calcium phosphate nucleation and mimic bone mineral maturation, also demonstrating attractive characteristics for bone tissue engineering [2,3,4]. The 45S5 coded glass synthesized by Hench et al. [2] and known as Bioglass contains P_2_O_5_–SiO_2_–CaO–Na_2_O. Similarly, the 13-93 composition (wt%), another bioactive glass used in biomedical applications, has 53% SiO_2_, 6 Na_2_O, 12 K_2_O, 5 MgO, 20 CaO, and 4 P_2_O_5_ contents [5]. Likewise, borate-based 13-93B3 bioactive glass has been developed by replacing SiO_2_ in the 13-93 bioactive glass composition with B_2_O_3_, and it has higher bioactivity compared to silicate-based bioactive glasses [6,7]. However, the primary disadvantage of borate glasses is their lower mechanical strength compared to their silicate-based counterparts. To overcome this limitation, the preparation of the bioactive glass matrix composites in the presence of two dimensional nanomaterials and also polymer coatings are commonly employed. Previously, the inclusion of pristine graphene [8,9,10,11], graphene oxide [12,13], and tungsten disulfide [14,15] in bioactive glasses has been reported. In general, the use of the aforementioned two dimensional materials in the glass network improved the mechanical properties of the composites.

Hexagonal boron nitride is a two dimensional material that is similar to graphene both physically and chemically and is therefore known as white graphene. It has the same crystallographic appearance; however, in contains boron and nitrogen atoms instead of carbon [16,17,18]. On the other hand, unlike graphene, it is an insulator. Studies in the field of biomedical applications related to hBN have revealed that boron nitride nanotubes are biocompatible and do not have a toxic effect [19,20]. On the other hand, there are also few studies reporting the cytotoxic influence of boron nitride nanotubes on certain cell types. Their toxicity was found to be highly dependent on the cellular accumulation enhanced for straight nanotubes [21]. For this reason, many composite structures, including boron nitride and, especially, hydroxyapatite–boron nitride composites, have been prepared and demonstrated as a scaffolding material for tissue engineering applications as well as a drug delivery vehicle [22,23,24]. In a former study, the influence of hexagonal boron nitride nanoparticles incorporated into a PCL and PCL-PLGA matrix, which was coated on the surface of borate bioactive glass scaffolds, have been investigated [25]. Results showed that the incorporation of hBN nanoparticles inside the polymer matrix improved the compressive strength of the bioactive glass composite scaffolds as well as their in vitro bioactivity and biocompatibility [25]. The function of boron nitride nanosheets as the reinforcing material on the mechanical strength of borosilicate glass matrix was also investigated by Saggar et al. [26]. Results indicated that fracture toughness and the flexural strength of the glass composites raised as a function of boron nitride concentration in the glass. Although the use of boron nitride-based systems in the biomedical field and the preparation of biocomposites have been studied, the inclusion of hBN nanoparticles directly inside the bioactive glass matrix on the structural, mechanical, and biological performance and drug-release behavior has not yet been published. This study aimed to fabricate borate-based bioactive glass scaffolds containing hexagonal boron nitride (0.1, 0.2, 0.5, 1, and 2 weight percent) nanoparticles for bone tissue engineering applications using the polymer foam replication method. In this context, the structural and morphological characteristics of hBN-containing bioactive glass composite scaffolds, compressive strength, in vitro mineralization in simulated body fluid, cytotoxicity against pre-osteoblast MC3T3-E1 cells, and the drug delivery properties of gentamicin and fluorouracil-loaded scaffolds were investigated.

## 2. Experimental Studies

### 2.1. Materials

In the study, 13-93B3 bioactive glass powders (5.5 Na_2_O, 11.1 K_2_O, 4.6 MgO, 18.5 CaO, 3.7 P_2_O_5_, 56.6 B_2_O_3_ wt.%) synthesized by the melt-quenching method (d_50_: 2.5 μm and a density of 2.5 g/cm^3^) were used. The hexagonal boron nitride (hBN) nanopowders (99.85%+ purity, 65–75 nm, density: 2.3 g/cm^3^) used in the preparation of the composites were obtained from Nanografi Nanotechnology, Ankara, Turkey. They contain 0.03% Fe_2_O_3_, 0.002% CaO, 0.04% MgO, and 0.1% B_2_O_3_ as an impurity. Anhydrous ethanol (≥99.9% purity) and ethyl cellulose (d = 1.14 g/mL) were purchased from Sigma-Aldrich (Steinheim, Germany) to be used in scaffold manufacture using the polymer foam replication method.

### 2.2. Porous Biomimetic Scaffold Manufacture

The polymer foam replication method, which was developed within the scope of another study, was used to manufacture three-dimensional bioactive glass scaffolds that mimic the cancellous bone [10,25]. In the method, poly(urethane) foams with a pore density of 60 pores per inch were cut to a diameter of 12 mm and a length of 35 mm and coated by dipping method using a homogeneously prepared bioactive glass-based suspension (40 vol.% bioactive glass powder, ethanol, 4% ethyl cellulose). Likewise, polymer foams coated with the same glass suspension, but also containing 0.1, 0.2, 0.5, 1, and 2 hBN nanoparticles by weight, were prepared and left to dry at room temperature and then heat-treated at 570 °C (1 h, heating rate < 1 °C/min below 350 °C) to remove the polyurethane foam in the structure and fabricate a dense three-dimensional bioactive glass scaffold.

### 2.3. Instrumentation

The morphological characteristics of the prepared glass scaffolds were examined with a stereo microscope (Nikon, SMZ745T, Tokyo, Japan) to observe the distribution of the additive nanoparticles with the bioactive glass and to determine the changes in the pore structure of the prepared scaffolds. In addition, total porosity measurement was performed using Archimedes’ principle to determine the porosity of the scaffolds.

FTIR spectroscopy (Thermo Scientific, Nicolet, IS20, Waltham, MA, USA) was used for structural analysis, and measurements were made by using an ATR module in the wavelength range of 550–4000 cm^−1^. In XRD analysis, the Malvern Pan-Analytical brand, Empyrean model diffractometer was used. A Cu-Kα X-ray tube was utilized in the measurement, and samples were analyzed in the range of 10°–90° at a scanning speed of 0.01°/min.

The effect of hBN nanoparticles used in the study on the mechanical properties of bioactive glass scaffolds was analyzed by compression test. Cylindrical bioactive glass scaffolds with a height of ~6–8 mm and a diameter of ~6 mm were used for the compression test. Measurements were carried out with a Shimadzu brand Autograph AG-IS model test device using a deformation rate of 0.5 mm/min. Measurements were performed for 5 different samples and results were averaged. Before mechanical testing, the contact surfaces of each sample were ground to produce parallel surfaces.

### 2.4. In Vitro Mineralization

The bioactivity of the samples was tested in simulated body fluid (SBF). For the preparation of the simulated body fluid, the protocol developed by Kokubo et al. [27] was followed. The chemicals NaCl, NaHCO_3_, KCl, K_2_HPO_4_.3H_2_O, MgCl_2_.6H_2_O, CaCl_2_, and Na_2_SO_4_ (Sigma-Aldrich, Steinheim, Germany) were dissolved in deionized water and buffered at a pH of 7.40 with tris(hydroxymethyl)aminomethane ((CH_2_OH)_3_CNH_2_) and 1 M hydrochloric acid (Fisher Scientific Inc., USA) at 37 °C. A total 500 mL of SBF per 1 g of sample was used and the scaffolds were disinfected with ethanol before immersion in SBF. Tissue scaffolds immersed in SBF were kept in an incubator at 37 °C for 7, 14, and 30 days. When the specified time expired, the scaffolds were removed from the incubator and left to dry after washing with deionized water and ethanol. The changes in the samples as a result of holding in SBF were examined using a scanning electron microscope (ZEISS, GeminiSEM 560) and FTIR spectroscopy under the conditions described previously.

### 2.5. Drug Delivery Studies

#### 2.5.1. Gentamicin

Gentamicin (Genta ampoule, İbrahim Etem Ulagay İlaç, İstanbul, Turkey, containing 80 mg/2 mL gentamicin, 124.8 mg in the form of gentamicin sulfate) was used as the first drug to investigate drug release behavior from bioactive glass composite scaffolds prepared in the study. Before the drug release studies, the samples were disinfected by soaking in ethanol, and then 50 microliters of Genta solution (40 mg/mL gentamicin) were dropped onto the sample surface with the help of a micropipette. After drying for 24 h, drug-loaded samples were soaked in a 5 mL of phosphate-buffered saline (PBS) solution and the amount of drug released into the PBS was measured with a UV–Vis spectrophotometer at 256 nm (Thermo Scientific, Evolution 201, Waltham, MA USA) for up to 96 h.

#### 2.5.2. Fluorouracil (5-FU)

For the 5-FU loading studies, bioactive glass composite scaffolds were immersed in a 5 mL of 5 mg/100 mL drug solution (5-FU, Sigma-Aldrich, Steinheim Germany), which is prepared in PBS (pH 7.4) for 48 h at 37 °C in a dark environment. In the adsorption study, at the end of 48 h, 2 mL of the drug solution mixture was taken, and the absorbance was measured at 266 nm with a UV–Visible spectrophotometer (Thermo Scientific, Evolution 201, Waltham, MA USA). Bioactive glass scaffolds were removed from the drug solution and dried at 40 °C for 48 h before release experiments.

For drug release studies, the drug-loaded bioactive glass scaffolds were immersed in 10 mL of PBS at pH 7.4. The release experiments were made at 37 °C under static conditions. For each time interval, 2 mL of the drug solution sample was taken and replaced with the same amount of fresh PBS solution. The absorbance values of the samples were recorded at 266 nm using the spectrophotometer, and the amount released from the calibration curve was obtained. Drug loading and delivery experiments were performed in triplicate and results were averaged. Through the experiments, the direct light contact of the drug solution was prevented and drug solutions were freshly prepared.

In the study, the drug release kinetics were also investigated. For this purpose, the obtained release profiles were analyzed using zero order, first order, and Higuchi kinetic models [28].

### 2.6. Cytotoxicity 

The in vitro cytotoxicity of the biomimetic scaffolds was examined using the osteoblastic cells (MC3T3-E1, Subclone-4, ATCC, CRL-2593, Manassas, VA, USA) using MTT (3-[4,5-dimethylthiazole-2-yl]-2,5-diphenyltetrazolium bromide) (Sigma-Aldrich, Steinheim, Germany) assay which is a colorimetric method used to understand the metabolic activity of living cells. Scaffolds were sterilized at 350 °C before cell culture experiments. Osteoblastic cells were cultured in a growth medium containing Alpha-Minimum Essential Medium with L-glutamine with 10% fetal bovine serum and 100 U/mL penicillin–100 mg/mL streptomycin. For this purpose, MC3T3-E1 cells (5 × 10^4^) were seeded onto each scaffold in the presence of 1.9 mL of culture media and cultured for 72 h at 37 °C in a 5% CO_2_ incubator. Following this, MTT solution was added and the cells were cultured for a further 4 h. The formazan crystals formed at this stage were dissolved by dimethyl sulfoxide (DMSO, Sigma-Aldrich, Steinheim, Germany). A multi-plate reader (Thermo-Scientific, Waltham, MA, USA) was used to measure the color change at a wavelength of 570 nm that is directly related to the amount of formazan. The morphology of the osteoblastic cells was observed after culturing with the glass scaffolds for 72 h using an optical microscope.

The statistical analyses for the MTT test results were carried out with Graph Pad Prism 5. Results were analyzed by using one-way ANOVA. Values with *p* ≤ 0.05 (*), *p* ≤ 0,01 (**) were considered statistically significant.

## 3. Results and Discussion

**Figure 1:**
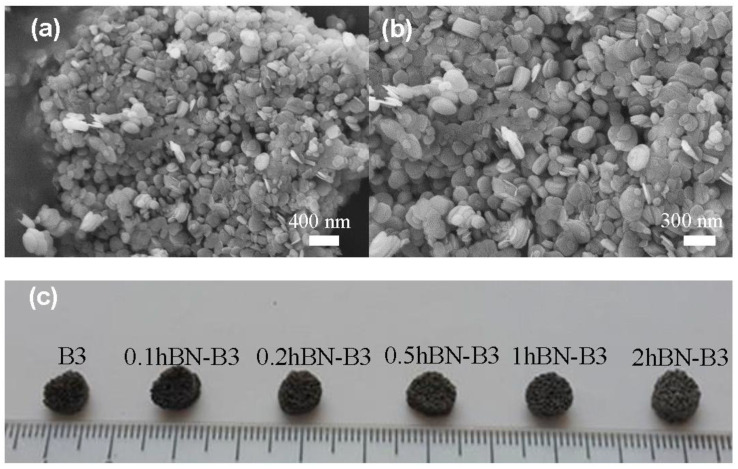
(**a**,**b**) SEM micrographs of the as-received hBN nanopowders; (**c**) digital image of the fabricated glass scaffolds.

The SEM images of hBN nanoparticles used in the study and the digital images of the fabricated bioactive glass scaffolds are given in Figure 1a–c. Based on the SEM micrographs and the report of the manufacturer company, the average particle size of the hBN nanoparticles is 65–75 nm, and it can be seen that they exist in a platelet structure with round morphology and also tend to agglomerate.

**Figure 2:**
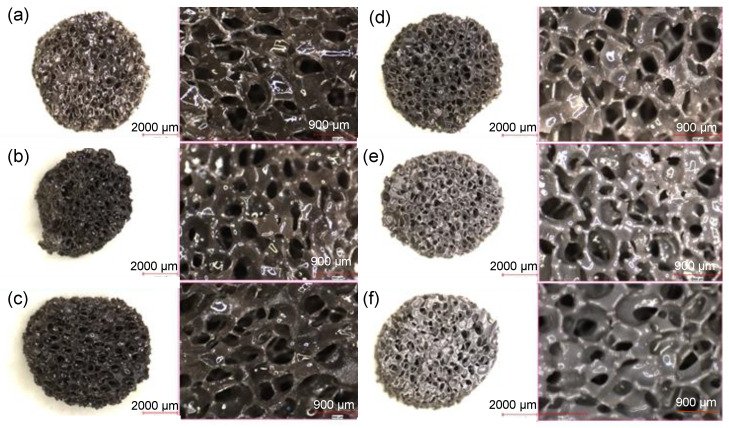
Optical microscope images of the (**a**) bare B3, (**b**) 0.1 hBN, (**c**) 0.2 hBN, (**d**) 0.5 hBN, (**e**) 1 hBN, and (**f**) 2 hBN-containing borate bioactive glass scaffolds. Low magnification image scale bar: 2000 µm; high magnification image scale bar: 900 µm.

The digital images of porous bioactive glass scaffolds (6 mm diameter, 3 mm height) produced using the polymer foam replication method and containing hBN nanoparticles at different volume ratios show no significant change in the morphology of the scaffolds at varying hBN concentrations. This result is also supported by the optical microscope images given in Figure 2. Optical microscope images demonstrate that the prepared scaffolds have an interconnected, open pore structure, and the addition of hBN does not change the pore structure significantly. It is understood from the optical microscope images given at high magnification that the average pore diameter is ~500 μm.

**Figure 3:**
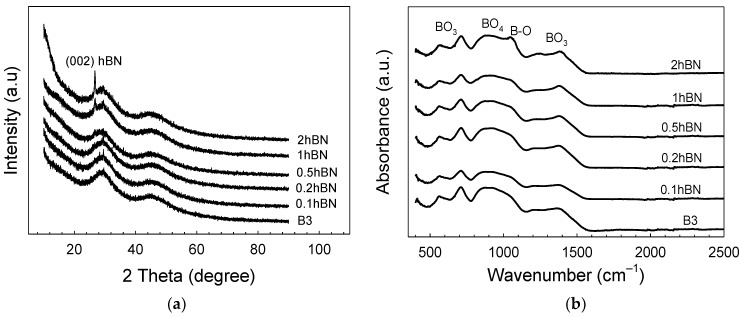
(**a**) XRD pattern; (**b**) FTIR spectra of the prepared bioactive scaffolds containing hBN nanopowders.

The XRD patterns of the prepared scaffolds after sintering at 570 °C are shown in Figure 3a. It can be seen that the glass samples subjected to heat treatment maintain their amorphous structure up to 0.5% hBN concentration. The characteristic peak formation of hBN in the structure is observed with bioactive glass samples at higher boron nitride concentrations. The intense peak observed in Figure 3a corresponds to the hBN peak of (2θ-27°) the 002 plane in the XRD pattern (JCPDS 034-0421). This characteristic peak is similar to the 2θ-26° peak seen in the XRD pattern of graphene. In addition, although the characteristic peak observed overlaps with the hydroxylated boron nitride (BNO) peak, it is known that BNO conversion occurs at 1000 °C [29]. It is known that the oxidation character of boron nitride nanostructures is affected by the specific surface area, and boron nitride nanocrystals (for 210 nm edge length, 270 nm thickness) maintain their thermal stability in the air up to 900 °C. [30]. In addition, the low-intensity peaks observed in the pattern at ~2θ-29° belong to B_2_O_3_ [31].

According to the FTIR spectra given in Figure 3b, it is seen that the inclusion of hBN nanoparticles in the bioactive glass structure does not cause a significant change in the molecular structure of the composite scaffolds. Accordingly, peaks at 1300–1500 cm^−1^ and 720 cm^−1^ wavenumbers are due to the presence of B_2_O_3_ groups. The broad peak in the 1300–1500 cm^−1^ wavenumber range belongs to the bending and stretching vibrations of the B–O–B bonds in the BO_3_ triple system and the low-intensity peak at ∼726 cm^−1^ is due to B−O−B linkages. On the other hand, the broad band in the range of 900–1100 cm^−1^ also belongs to the stretching vibrations of BO_4_^−^ groups in the structure [32]. In the spectrum of the 2hBN-B3 glass, the shoulder observed at 1100 cm^−1^ corresponds to the B–O–H in-plane bending [33].

**Figure 4:**
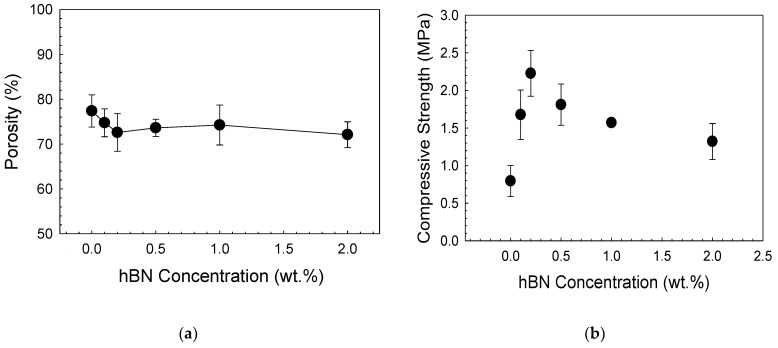
Graphs showing (**a**) the total porosity and (**b**) the compressive strength of the composite scaffolds.

In Figure 4a, according to the graph showing the total porosity values of the scaffolds, it was determined that there was a slight decrease in the porosity values with increasing hBN concentration. While the mean porosity value of the scaffolds without hBN was 77 ± 3.6%, this value was measured as 72 ± 2.8% in the scaffold with the highest hBN concentration.

In Figure 4b, the compressive strength values of the scaffolds are given. Accordingly, while the compressive strength value of the 13-93B3 scaffold without additives was 0.79 ± 0.2 MPa, the compressive strength of the sample containing 0.2% hBN was measured to be 2.22 ± 0.3 MPa. A decrease in compressive strength values was observed at hBN concentrations higher than this value presumably due to the agglomeration of hBN nanopowders added to the structure at high concentrations. 

In a previous study by Turk and Deliormanlı [10], the compressive strength of 13-93B3 bioactive glass scaffolds prepared using the polymer foam replication method containing graphene nanopowders at different concentrations (1, 3, 5, and 10 wt%) was investigated. The results showed that the highest compressive strength value was obtained in the sample containing 5% graphene as 1.86 ± 0.7 MPa. In the current study, the maximum compressive strength value was obtained for the hBN-containing 13-93B3 bioactive glass scaffolds at 0.2% hBN concentration, and the strength of these scaffolds is approximately 19% higher than that of graphene-bioactive glass scaffolds. The special layer-stacking structure of hBN, the partial ionicity of boron and nitrogen atoms, and the polarity of the orbitals in the structure may influence the observed increase in the compressive strength of the scaffolds. Due to the partial ionicity of the B and N atoms in the hBN structure, the layers are aligned by the overlapping of the positive B and negative N atoms. In this particular case, the hBN atoms have an AÁ stacking structure, while the graphite structure has an AB (Bernall stacking) stacking. In this case, only some of the carbon atoms are located directly above or below the neighbor. Unlike graphene, in hexagonal boron nitride, the layer thickness does not affect the mechanical properties much. As the layer thickness increases in the graphene structure, the deviation of the mechanical property values is related to the inhomogeneous deformation and stacking structure. These mentioned factors cause the shift of the interlayer and energy loss during the loading and unloading cycle. The main reason for this difference in graphene is the spontaneous sliding of the graphene layers on the graphene surface due to the negative increase in shear energy as a result of large in-plane stress and out-of-plane pressure applications and warped layer (AB) stacking. This is because of the overlapping 2Pz orbitals in the graphene structure. On the other hand, the more polar orbitals in hBN become localized in the same stress condition to positively increase the sliding energy barrier, thus making the hBN resistant to interlayer shifting [34,35,36,37,38,39]. In the current study, the improvement obtained in the compressive strength of bioactive glass-based scaffolds in the presence of hBN nanoparticles may be attributed to the uniform dispersion of the nanoparticles in the glass matrix and the stress transfer between the nanoparticles and matrix. A higher level of nanoparticle loading presumably reduced the load transfer between matrix and filler due to the agglomeration of nanoparticles, which in turn caused a decrease in the compressive strength.

In another study [40], it was reported that boron nitride nanolayers added to the akermanite matrix improved the mechanical strength of akermanite. In that study, the compressive strength of akermanite-boron nitride nanolayer composites increased with 0.5% and 1% boron nitride additives by weight, while the compressive strength decreased at higher concentrations.

**Figure 5:**
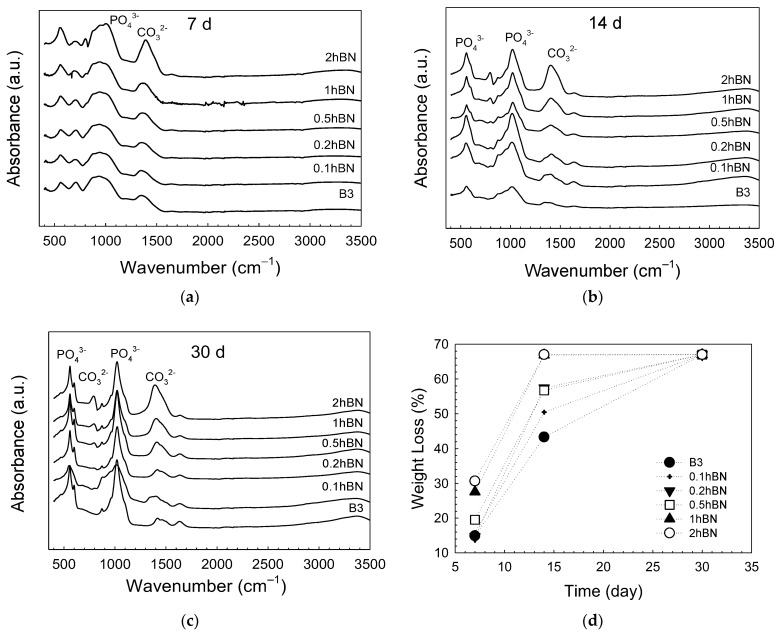
FTIR spectra of the SBF treated bioactive glass scaffolds for (**a**) 7d, (**b**) 14 d, and (**c**) 30 d; (**d**) graph showing the weight loss of the scaffolds as a function of SBF immersion time.

The FTIR spectra of bioactive glass scaffolds kept in SBF for 7, 14, and 30 days are given in Figure 5a–c. When the results of the SBF-treated (7 days) scaffolds were examined, no significant difference was observed in the spectra of the scaffolds at low hBN concentrations, whereas the formation of PO_4_^3−^ (at 550 cm^−1^ and 1000 cm^−1^) and CO_3_^−^ (at 1389 cm^−1^) groups starting from 1% hBN were observed. This is particularly evident in glass samples containing 2% hBN. When the scaffolds kept in SBF for 14 days were examined, the peaks representing hydroxyapatite formation on the surfaces of the samples were seen on the spectrum. Accordingly, the peak at 1022 cm^−1^ was assigned to the PO_4_^3−^ group v_3_ vibration, and the peak at 554 cm^−1^ corresponded to the v_4_ vibration of the same group. The split peak at 550 cm^−1^ and 604 cm^−1^ observed in the glass samples kept in SBF for 30 days belongs to the bending mode of orthophosphate and demonstrates that hydroxyapatite is formed in the structure. The peak observed at 964 cm^−1^ represents the PO_4_^3−^ v_1_ vibrations and the peaks at ~874 cm^−1^ and ~1400 cm^−1^ may be due to the presence of the CO_3_^−^ group [41,42]. Results revealed that peak intensities representing crystalline hydroxyapatite formation increased as a function of immersion time in SBF.

In general, the presence of hBN nanoparticles appeared to enhance the HA-forming ability of bioactive glass starting from certain concentrations, presumably due to their high surface area. Similarly, in a different study, hydroxyapatite precipitation was reported on the surface of boron nitride nanoparticles in 5 days when immersed in simulated body fluid [43]. This result supports the increase in bioactivity seen in bioactive glass scaffolds containing hBN nanoparticles.

The weight loss values of the fabricated bioactive glass scaffolds after soaking in SBF at 37 °C are given in Figure 5d. Accordingly, it was observed that the bare borate glass scaffolds kept in SBF for 7 days lost approximately 15% of their original weight, while this value was calculated as ~31% in the scaffolds containing 2% hBN. At the end of 30 days, it was observed that all of the glass scaffolds under investigation lost 67% of their weight. It was observed that the percentage of weight loss increased with increasing hBN concentration. This behavior may be correlated with the loose network of the borate glasses. Unlike silica, the coordination number of boron prevents the full formation of the 3D network structure, causing the boron-based glass to have lower chemical stability [43]. It is also known that ions such as Na^+^, K^+^, Mg^2+^, and (BO_3_)^−3^ dissolve in solution and all CaO in the glass reacts with phosphate ions in SBF to form HA and the theoretical weight loss of fully transformed 13-93B3 bioactive glass scaffolds is 67% [3,44].

**Figure 6:**
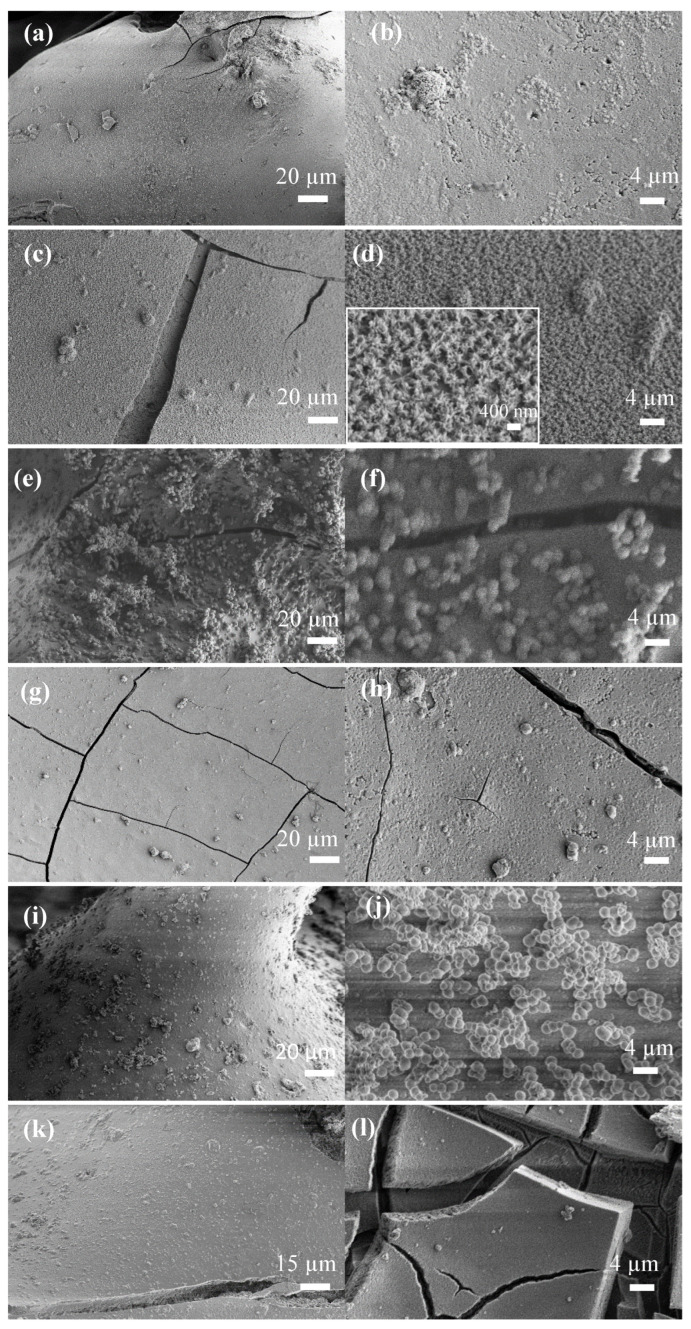
SEM micrographs of the SBF-treated bioactive glass scaffolds for 7 d (**a**,**b**) B3; (**c**,**d**) 0.1 hBN-B3; (**e**,**f**) 0.2 hBN-B3; (**g**,**h**) 0.5 hBN-B3; (**i**,**j**) 1 hBN-B3; (**k**,**l**) 2 hBN-B3.

**Figure 7:**
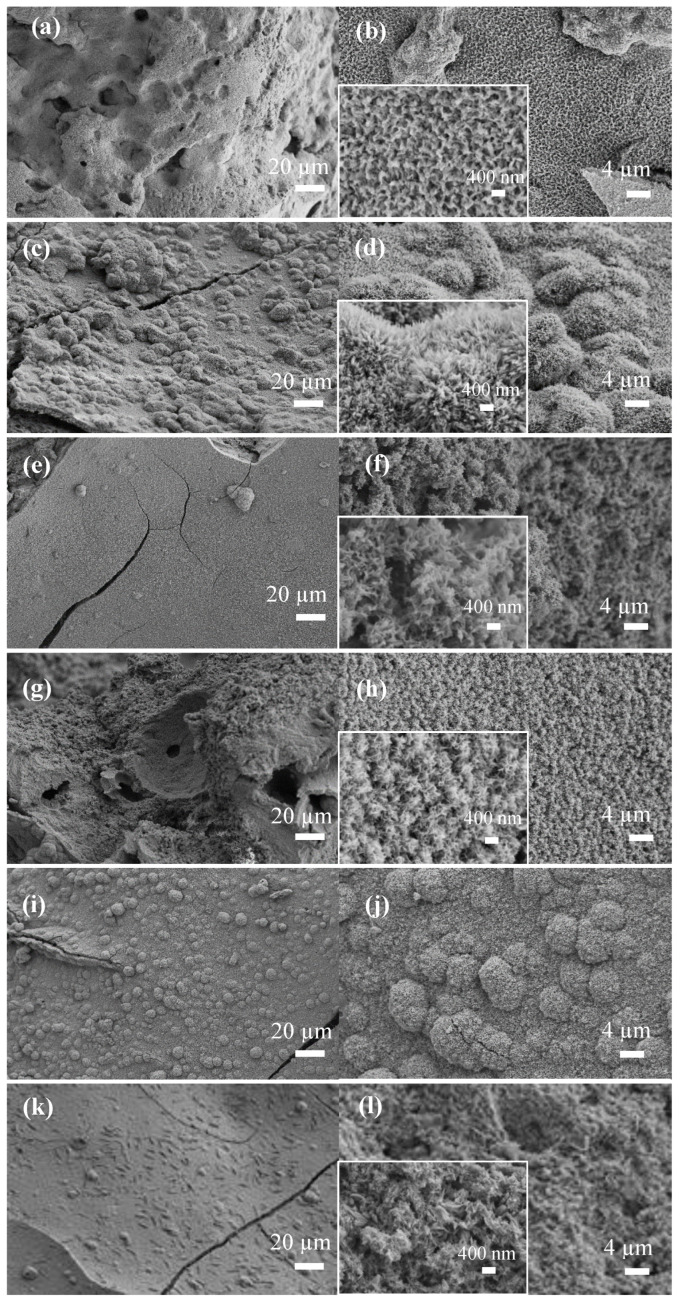
SEM micrographs of the SBF-treated bioactive glass scaffolds for 30 d (**a**,**b**) B3; (**c**,**d**) 0.1 hBN-B3; (**e**,**f**) 0.2 hBN-B3; (**g**,**h**) 0.5 hBN-B3; (**i**,**j**) 1 hBN-B3; (**k**,**l**) 2 hBN-B3.

The SEM images of glass scaffolds kept in SBF for 7 days and 30 days are given in Figure 6 and Figure 7, respectively. Accordingly, it is understood that a new substance formation occurred on the surface of the scaffolds, which were kept in SBF for both 7 days and 30 days. At the end of 30 days, HA formation increased significantly and formed a thick layer on the surface of the scaffolds. Plate-like HA formations came together to form spherical aggregates. It was determined that the second phase material observed in the micrographs was compatible with the HA morphology [45,46].

**Figure 8:**
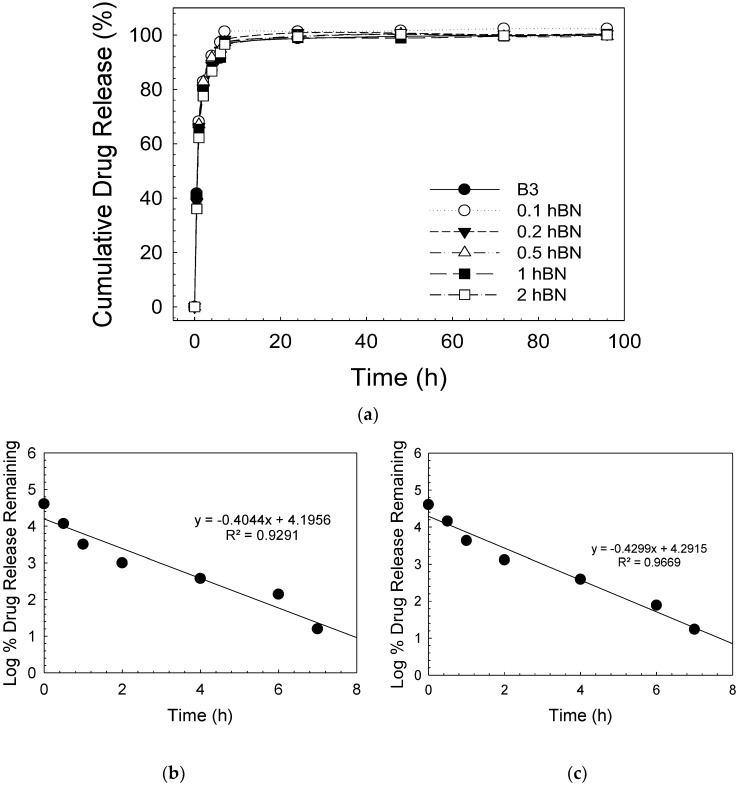
(**a**) Cumulative gentamicin sulfate release curve; graphs showing the first order drug release for (**b**) bare B3, (**c**) 2 hBN-B3.

The results of gentamicin release experiments from bioactive glass scaffolds containing hBN are demonstrated in Figure 8. In this study, drug delivery from the scaffolds loaded with gentamicin sulfate was monitored for up to 96 h. The results revealed that the gentamicin release from the scaffolds was very rapid and the cumulative release amount reached 100% in up to 24 h. This may be attributed to the weak physical adsorption of the gentamicin sulfate to the glass surface. Drug release kinetic studies revealed that the highest correlation coefficient (R^2^) value among gentamicin release profiles was obtained in the first order model (see Table 1). The logarithm of the percentage of the drug remaining in this kinetic model [47] versus the time (hour) plot gives a straight line. It can be seen that the drug release behaviors of the bare and hBN-containing bioactive glass samples (at all concentrations) were similar.

**Figure 9:**
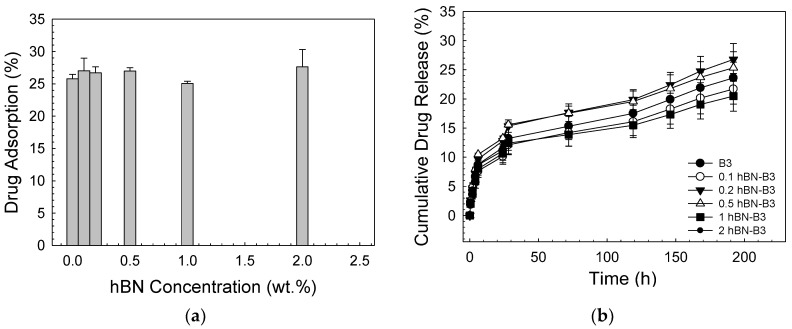
(**a**) 5-FU adsorption curve; (**b**) cumulative 5-FU release curve for studied bioactive glass scaffolds.

**Figure 10:**
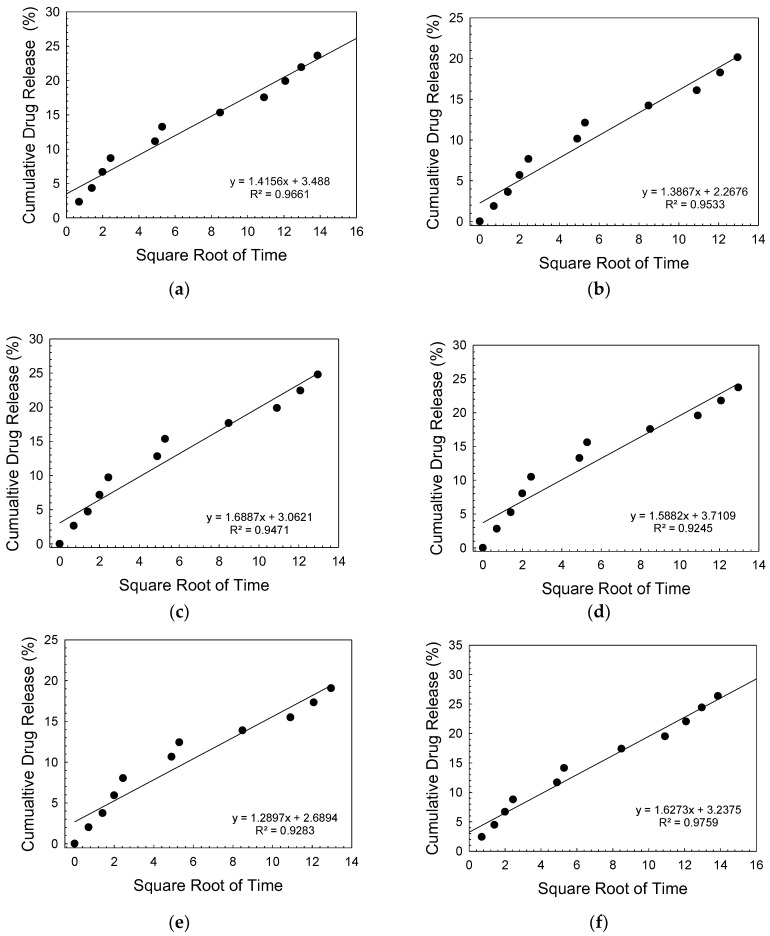
Graphs showing the Higuchi model 5-FU release for (**a**) bare B3, (**b**) 0.1 hBN-B3, (**c**) 0.2 hBN-B3, (**d**) 0.5 hBN-B3, (**e**) 1 hBN-B3, and (**f**) 2 hBN-B3.

The other drug tested in the study was fluorouracil. The antimetabolite drug fluoropyrimidine 5-fluorouracil is frequently used to treat cancer. 5-FU has anticancer properties, since it inhibits thymidylate synthase and integrates its metabolites into RNA and DNA [48,49,50]. Figure 9 demonstrates the results of the fluorouracil loading and the release experiments into a PBS medium. Accordingly, the drug adsorption percentage to the bioactive glass scaffolds was in the range of 25% to 30%. Drug release experiments showed that after 24 h, the cumulative drug release amount was calculated to be 11% to 13%. After 196 h immersion in PBS, 20 to 26% of the drug was released from the scaffolds. The difference obtained in the cumulative release rates between gentamicin and the 5-FU may be attributed to the chemical structure of the drugs and the loading method followed in the experiments. In a past study, Dehaghani et al. [51] investigated the encapsulation of 5-FU into carbon nanotubes and boron nitride nanotubes (BNT) theoretically. Results revealed that due to the raised van der Waals contact energy between the drug and the BNT, 5-FU was adsorbed into the cavity of the BNT more quickly than the CNT. Similarly, the electrical response of BN nanocones to 5-FU was studied by Wang et. al. [52] using density functional theory. Results indicated that the boron nitride nanocones may be suitable candidates for the detection of 5-FU and can be utilized in electronic sensors. In the current study, the drug release kinetics studies for 5-FU showed that the release of the drug followed Higuchi kinetic model (R^2^ = 0.97 and 0.98 bare and 2% hBN-containing glass scaffolds, respectively), which describes the diffusion-based delivery process from a porous vehicle (Table 2). Figure 10 depicts the drug release graphs fitted by the Higuchi kinetic model. El-Kady et al. [53] also reported that 5-FU release from silicate-based sol-gel-derived bioactive glass nanoparticles was fitted using the Higuchi model (square root of time) and followed by a diffusion-controlled release mechanism.

**Figure 11:**
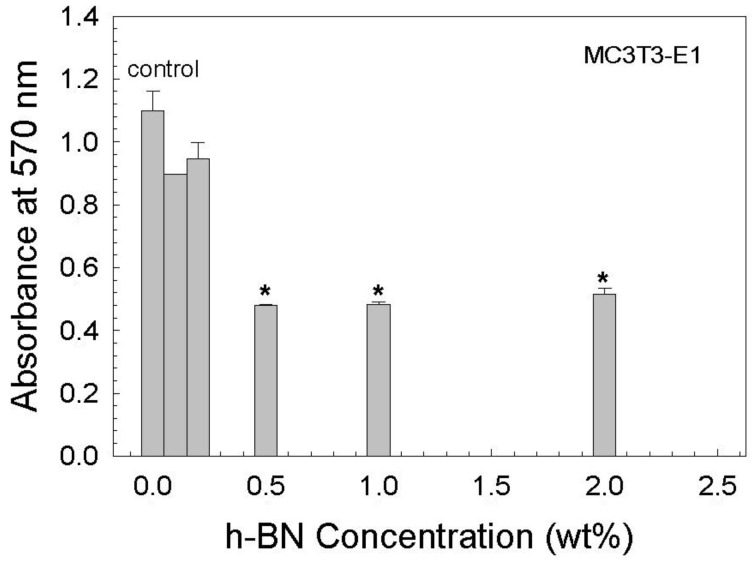
Graph showing the MTT assay results after incubation for 72 h. * indicates the statistical significance, *p < 0.05*.

The results of the MTT assay, which was made to understand the cytotoxicity of the fabricated samples, are given in Figure 11. Accordingly, starting from a 0.5% hBN content, a significant decrease (~50%) was observed in cell viability after 72 h. However, cell viability was calculated to be 86% for 0.2% hBN-containing glass scaffolds under the same conditions. This may be attributed to the increase obtained from the in vitro degradation of the glass scaffolds as the hBN concentration increased. Recently, it was seen that when they are used as a coating material inside a polymeric matrix over borate-based 13-93B3 bioactive glass scaffolds, hBN nanoparticles did not show any toxic effect for the same type of cells [25]. However, in the current study, hBN nanoparticles were embedded directly inside the glass matrix, and following the degradation of the glass in the cell culture medium, 2D nanoparticles may diffuse into the cell membrane more easily. Similarly, a recent study by Khalid et al. [54] reported the toxicological effects of boron nitride nanotubes. Results showed that the cytotoxicity of boron nitride nanotubes was higher in HeLa cancer cells (80%) compared to the HEK-293 normal cells (60%) for 48 h incubation.

**Figure 12:**
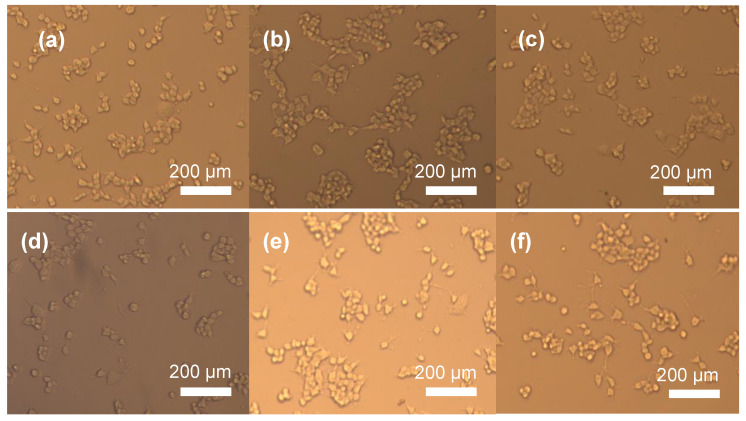
Optical microscope images of the MC3T3-E1 cells after incubation for 72 h in the presence of composite bioactive glass scaffolds containing (**a**) 0, (**b**) 0.1%, (**c**) 0.2%, (**d**) 0.5%, (**e**) 1%, and (**f**) 2% hBN nanopowders. Magnification at 100×.

Figure 12 shows the optical microscope images of the osteoblastic MC3T3-E1 cells after incubation for 72 h in the presence of fabricated bioactive glass composite scaffolds. Based on the optical microscope images there was no significant difference in cell morphology depending on the hBN concentration in the glass matrix.

## 4. Conclusions

Within the scope of this study, biomimetic borate bioactive glass scaffolds containing hBN nanopowders were produced and in vitro characterizations were carried out. According to XRD analysis results, it was seen that hBN generally remained stable in the bioactive glass matrix. The applied mechanical test results showed that the hBN nanoparticle addition (depending on the concentration) improved the compressive strength of the bioactive glass scaffolds. In vitro bioactivity studies in SBF indicated that the incorporation of hBN improved the hydroxyapatite-forming ability of bioactive glass scaffolds. According to the results of drug release experiments of antibiotic-loaded scaffolds in PBS medium, it was understood that all scaffolds showed burst drug release behavior, presumably due to the rapid degradation of 13-93B3 glass and weak interaction of the drug molecule with the glass surface. It was observed that the drug release kinetics of the scaffolds fit the first order kinetic model. On the other hand, the cumulative release of 5-FU from the scaffolds was measured to be 20% to 26% after 196 h. Fluorouracil showed sustained release behavior from the borate glass scaffolds and the release kinetics were fitted by the Higuchi model. In vitro cytotoxicity experiments revealed that the presence of hexagonal boron nitride nanopowders in a bioactive glass matrix up to 0.2% did not cause any toxicity in mouse calvarial pre-osteoblast cells after 72 h of incubation.

## Figures and Tables

**Table 1:** Gentamicin sulfate release kinetics model parameters. K_0_, K_1_, and K_H_ are zero order, first order, and Higuchi model rate constants. t is time, and C_0_ is the initial concentration of the drug.

Sample	Zero Order $C_{t}=C_{0}+K_{0}.t$		First Order $logC=logC_{0}-K_{1}.t/2.303$		Higuchi $Q=K_{H}\sqrt{t}$	
	R^2^	K_0_	R^2^	K_1_	R^2^	K_H_
B3	0.6478	10.024	0.9291	0.4044	0.8682	33.372
0.1 hBN-B3	0.6729	10.901	0.9809	0.5964	0.8855	35.956
0.2 hBN-B3	0.6754	10.595	0.9526	0.5013	0.886	34.895
0.5 hBN-B3	0.6462	10.389	0.9501	0.4641	0.8681	34.626
1 hBN-B3	0.6671	10.229	0.9357	0.4301	0.8819	33.82
2 hBN-B3	0.7027	10.676	0.9669	0.4299	0.9041	34.824

**Table 2:** 5-FU release kinetics model parameters. K_0_, K_1_, and K_H_ are zero order, first order, and Higuchi model rate constants. T is time, and C_0_ is the initial concentration of the drug.

Sample	Zero Order $C_{t}=C_{0}+K_{0}.t$		First Order $logC=logC_{0}-K_{1}.t/2.303$		Higuchi $Q=K_{H}\sqrt{t}$	
	R^2^	K_0_	R^2^	K_1_	R^2^	K_H_
B3	0.896	0.0923	0.9138	0.0011	0.9661	1.415
0.1 hBN-B3	0.893	0.0866	0.9097	0.001	0.9669	1.331
0.2 hBN-B3	0.887	0.1048	0.9079	0.0012	0.9632	1.613
0.5 hBN-B3	0.59	0.0951	0.8814	0.0011	0.9487	1.476
1 hBN-B3	0.859	0.782	0.8767	0.0009	0.948	1.214
2 hBN-B3	0.908	0.1063	0.9268	0.0013	0.9759	1.627

## Data Availability

Not applicable.
